# Explaining the Evolution of Warning Coloration: Secreted Secondary Defence Chemicals May Facilitate the Evolution of Visual Aposematic Signals

**DOI:** 10.1371/journal.pone.0005779

**Published:** 2009-06-03

**Authors:** Jostein Gohli, Göran Högstedt

**Affiliations:** Department of Biology, University of Bergen, Bergen, Norway; American Museum of Natural History, United States of America

## Abstract

Several pathways have been postulated to explain the evolution of warning coloration, which is a perplexing phenomenon. Many of these attempt to circumvent the problem of naïve predators by inferring kin selection or neophobia. Through a stochastic model, we show that a secreted secondary defence chemical can provide selective pressure, on the individual level, towards developing warning coloration. Our fundamental assumption is that increased conspicuousness will result in longer assessment periods and divergence from the predators' searching image, thus reducing the probability of a predator making mistakes. We conclude that strong olfactory signaling by means of chemical secretions can lead to the evolution of warning coloration.

## Introduction

The evolution of warning coloration [1] has continued to be a persistent problem for evolutionary biologists. Signals used by aposematic prey increase conspicuousness and/or distinctiveness [2] and will increase the initial probability of attack from predators [3], [4]. If predators are inexperienced, they must sample the aposematic prey to learn the association between the signal and the level of profitability. When aposematism first evolved, all predators were inexperienced and the population of aposematic prey would have been very small. Sampling (killing) would likely have led to an early extinction of this fragile population. A way to circumvent this fundamental problem is to postulate the use of reliable signals, thus removing the need for sampling and learning. It would therefore serve an aposome well to mediate its unpalatability via odorous secretions which can function in such a manner, thus avoiding close contact with the predator [5]. By causing irritation and/or pain when inhaled, such chemicals can give a reliable signal relating to the level of defence. It is difficult to imagine a predator who chooses to attack prey which makes its eyes burn, and causes pain in its respiratory system. In such a case, the chemical secretion is both a signal and a secondary defence component.

Olfactory aposematism [6] has not gone unnoticed by biologists. Both Cott [7] and Rothschild [8] discussed the pungent odours emitted by several aposomes. Cott suggested that odours emitted by aposomes may serve as a noxious defence, in addition to being a warning signal. Rothschild also gave examples of odours which themselves are clearly noxious. Prudic [9] and Eisner, Eisner, and Seigler [10] provides a more recent discussion of smelly defensive secretions. However, none of these discusses the potential effects of such secretions on the evolution of warning coloration.

We explore the possibility that chemical secondary defence could have set the stage for the evolution of warning coloration. By showing that a reliable chemical signal would select for increased visual conspicuousness, we provide a novel explanation to the evolution of visual aposematism. Speed and Ruxton [11] discussed the role of physical secondary defences in the evolution of aposematism. We modify their simulation model to analyse our hypothesis using a stochastic model.

## Methods

### The Model

The following is a model of optimal prey defence and signaling based on the factors visual conspicuousness (VC) and olfactory signal/defence (OSD). We define OSD as a released chemical toxin that acts both as a secondary defence agent and as an olfactory signal. This secondary defence can cause pain/irritation in the eyes and/or respiratory system, and may even irritate/damage the nervous system. For simplicity, we assume a linear relationship between signal strength and defence strength (a strong defence can not produce a weak signal and vice versa).

Our model assumes no initial aversion towards aposematic traits or conspicuousness, i.e. neophobia and/or dietary conservatism are not operating. Our model would work well with neophobia and/or dietary conservatism present, but it is not dependent on it. It is not possible to identify whether neophobia was present before aposematism or if it is an evolutionary response to aposematism, therefore a model explaining the evolution of aposematism can not build on the assumption of a neophobic response or similar aversions. We discuss the outcome of single interactions between predator and prey, altering only the variables VC and OSD. VC is given the interval [1.1–1.85] and OSD is given the interval [0.1–0.85]. These intervals could be standardized and modified by constants. However, we feel that this would only act to conceal the mechanics of our model. Both variables are dimensionless and are based around the population mean values. In the eventual empirical testing, both variables can be expressed in distance. Values of VC correspond to the distance at which predators locate the prey through sight. Similarly, the ODS value describes the distance at which the predator discover the prey by olfaction. Increased visual conspicuousness and odour intensity would of course result in detection at greater distances. Thus, visual and olfactory conspicuousness are *directly* correlated to our values. Importantly, the value for ODS also describes the strength of the deterrent effect of the signal. When an olfactory signal is “stronger”, more toxin reaches the recipient which results in a stronger deterrence. In nature numerous variables other than the signal strength affect the distance at which the signal is functional, wind affects olfactory signals and vegetation density affects visual signals for instance. Such complicating factors have not been included in our model. The model describes interactions between totally naive predators and totally egocentric prey (no kin selection).

We include four probabilities in our model:

- Pd = Probability of being detected (where k is a constant)
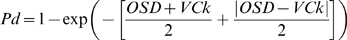

- Pa = Probability of being attacked once detected
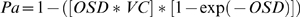

- Pk = Probability of being killed once attacked
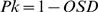

- Φ = Total probability of being killed
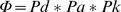

Pd is based on the one of the two variables (OSD and VC) exhibiting the largest value (the interval for VC is modified by *k*). If, for instance, prey is highly visually conspicuous, a weak olfactory signal will have no effect on Pd. On the other hand, should the prey be visually cryptic, a strong olfactory signal will be the governing variable. The intervals are modified in a way that grants VC the most power over Pd. Although this is not always the case (based on different predators' perceptive abilities and different habitats), we conclude that this is the most realistic scenario.

**Figure 1:**
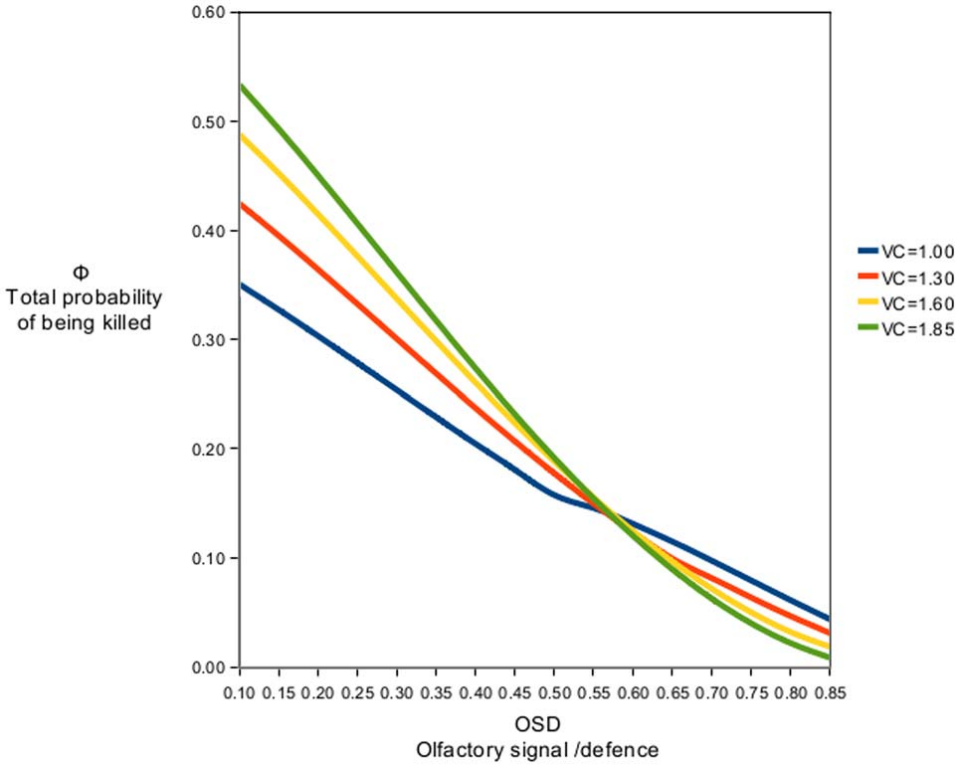
Visual and olfactory components and total probability of being killed. Different fixed values for VC (visual conspicuousness) are plotted against OSD (olfactory signal and defence) values, showing variation in the total probability of being killed (Φ). Selective forces acting on conspicuousness undergo a shift when defence levels reach a critical value (point of intersection). Our model predicts that maximum conspicuousness is the best strategy when the individuals are maximally defended through OSD.

We treat the probability of being attacked after detection (Pa) as solely dependent on the variables OSD and VC. The probability of attack will be reduced by increasing OSD values, because of OSD's chemical defence component. In the model, general conspicuousness (visual conspicuousness (VC) combined with the olfactory signal component of OSD) functions to enhance the effects of the defence, which is expressed through (OSD*VC), and is dependent on the intervals given for OSD and VC. A higher level of conspicuousness with no defence (OSD) will result in a higher Φ (see intercept values for different VC values in Figure 1). However, an individual with a high OSD value will benefit from the longer assessment period provided by higher general conspicuousness (Figure 1). We explain this fact by the following assumptions: the general conspicuousness ties into the length of the assessment period, because predators will detect prey items from longer distances when they are highly conspicuous. Since a common assumption is that predators may make mistakes, we correlate the assessment period/general conspicuousness to the probability of making a mistake. As the predator will be focused on the prey while moving down a gradient of noxious chemical defence, the prey's low profitability will be highlighted, and mistakes will be less probable. The length of this gradient is tied to general conspicuousness. Prey with a low VC value may be detected through the signal component of OSD or through visual cues (although the prey is visually cryptic), resulting in a shorter detection distance and assessment period. We base this on the assumption that visual signals work over greater distances than olfactory signals. In spontaneous attacks with short assessment periods, predators may not register the level of secondary defence, fatally injuring or killing the defended prey. Our assumption regarding the effect of the assessment period is supported by Gamberale-Stille [12], who showed that decision time is important in determining attack probability in both naïve and experienced predators.

There is a second immediate positive effect of developing increased visual conspicuousness together with chemical secondary defence. Increasing conspicuousness is a sure way of becoming visually distinct from other cryptic prey [13], and no longer coinciding with the predators' searching image. When a prey animal is visually identical to a predators' searching image, a more intense chemical OSD should be required to deter the predator. We are not describing neophobia, simply a divergence from the searching image of predators, which naturally goes hand in hand with divergence from the maximum-crypsis strategy. Many predators will react to prey coinciding with its searching image with immediate attack, which, as previously discussed, will increase the probability of making mistakes.

We assume that OSD is correlated to all forms of chemical defence, thus also affecting the probability of being killed after attack (Pk). This is based on the taste component, where a defended individual has a higher probability of being rejected [14]. The chemical secondary defence component should also reduce the intensity of the attack, further lowering Pk.

## Results

The classical problem of the evolution of warning coloration is described by OSD*i* and high VC values. An increase in conspicuousness with OSD at a fixed value of 0.1 will increase the total probability of being killed when predators are naïve (Figure 1; different VC-values, OSD = 0).

As one would expect, an increase in chemical secondary defence decreases the total probability of being killed (Figure 1). Because of the secondary defences' odour, it also increases the probability of being detected. However, considering the reliable nature of the signal component of OSD, this increased “olfactory conspicuousness” is profitable. Further increasing the general conspicuousness of the prey through a higher VC will be profitable once OSD reaches a certain level. Figure 1 shows different fixed values for VC combined with increasing values for OSD. The model shows that, given a critical value of OSD, it is also profitable to display warning coloration. This critical value differs slightly for different VC values. The curves also show how VC acts to enhance the effect of OSD. The fact that all values of OSD reduce the total probability of being killed (Φ) (illustrated by the VC = 1 curve) before warning coloration becomes profitable clearly shows an evolutionary path for the development of warning coloration.

Our results and predictions should be possible to test empirically, since the factors VC and OSD can be easily manipulated experimentally.

## Discussion

Our model indicates that warning coloration may be profitable when it is coupled to a secreted defence chemical. This statement holds true when predators are totally naïve and exhibit no neophobia. The benefits to defended prey are applicable at the individual level. These facts set our results apart from many of the other attempts to explain the evolution of warning coloration.

In calculating Pa, we make the assumption that general conspicuousness increases the assessment period and that this gives increased protection to prey with certain levels of defence (Figure 1). Higher conspicuousness makes the prey easier to spot, and therefore increases the possibility that the predator is further away from the prey when it is detected. The predator will be focused on the prey for a longer time period when it has to approach it from a distance. Moving towards the prey, the predator will move down a gradient of noxious chemical defence. During this time, the effects of the defence will become gradually more apparent. Once the predator reaches the prey, the defence will be at its maximum effect. This could be considered a form of “intensive learning”; while keeping its focus on the prey and moving towards it, the unpleasantness caused by the defence chemical increases, a fact resulting in the predator learning the association between defence and prey. While it is unlikely that a predator will choose to attack prey which is defended in this way regardless of visual conspicuousness, the increased assessment period and “intensive learning” will result in fewer mistakes. We base this on the assumption that spontaneous attacks are more prone to result in mistakes than attacks after assessment.

Since the prey may not be aware that a predator is approaching, it might pay to secrete these chemicals continuously. This proposal of continuous secretion might be controversial since insects seen in nature today more often control their defensive secretions and do not release them unless they are first disturbed. However, this apparent problem is created on false grounds because the selective regimes which are prevalent in nature today are not at all similar to those that ruled when aposematism first evolved. If constantly secreted defence compounds result in increased protection against naive predators (pre-aposematism), it also follows that this would have been the adopted strategy for prey animals. When warning coloration had been fixated in the prey population *and* learned by the predators/imprinted in the predator psyche, there would no longer be sufficient reason to continuously secrete the defence compounds. In fact, when the selective pressure created by the naive predators was reduced, the cost of continuous secretion would likely have resulted in a selective pressure towards increased control of secretion. This could explain why we do not often observe prey constantly secreting defensive compounds today.

When prey is conspicuous and chemically defended, predators may learn the association between defence and signals without sampling. When learning has taken place, it may be profitable for the prey to reduce the amount of defence chemical released. Even though naïve predators are not a problem only for the initial evolution of aposematism, the cost of secreting excessive amounts of defence chemicals may outweigh the cost of the odd naïve predator, as mentioned. A simple cost-benefit argument illustrates this point. Initially when all predators are naïve, prey has to be more or less constantly defended, but as predators associate defence with prey traits (for instance visual signals), it no longer pays prey individuals to invest maximally in defence compound secretion. Instead, there will now be a selective benefit of maximizing distant, i.e visual, recognition, paving the way for the evolution of visual/acoustic signals. However, there will always be inexperienced, young predators, a fact explaining the preservation of the reliable olfactory signal.

Conspicuousness gives increased protection for certain values of OSD in our model, but visual signals can give additional advantages beyond those described by our model, once predators have learned the association between signals and defence. Visual and olfactory signals are very different in nature and are affected differently by environmental factors. Once olfactory aposematism is established, based on a reliable signal (OSD), it will probably pay to advertise profitability with more than one signal, taking into consideration the effects of multimodality [15], [16] and the fact that different signals work differently and on different scales. Visual signals may, in certain habitats, be more far-reaching than olfactory signals. Such an increase in signaling distance should decrease the number of close encounters with predators (if the visual aposematic trait is familiar to the predator), which will be stressful no matter what the outcome. Warning coloration could also have evolved based on these advantages once olfactory aposematism had been established. This would not be dependent on our assumptions in calculating Pa (fewer mistakes through a higher assessment period).

As most visual aposomes are insects and their main predators often are birds, a quick word on the olfactory capabilities of birds is in order. Albeit an old and common misconception, the belief that birds are “poor smellers” or that they do not rely heavily on olfaction, is a misconception nontheless. Experimental works on aposematism have shown that odour is an important cue for chickens (*Gallus gallus domesticus*) when foraging [15], [16]. Other works have discussed and provided evidence showing that smell is much more important in birds than previously thought [17], [18].

Through our model we have shown, that given a reliable olfactory signal, visual conspicuousness is a profitable strategy. The element of reliability removes the problem of sampling/killing by naïve predators, making it possible for visual signals to accompany the olfactory element. We conclude that olfactory signals/secreted toxins provide a solution for the evolution of visual aposematic traits.

## References

[1] Wallace AR (1867). Proc Entomol Soc Lond (4 March).

[2] Merilaita S, Ruxton GD (2007). Aposematic signals and the relationship between conspicuousness and distinctiveness.. J Theor Biol.

[3] Fisher RA (1930). The genetical theory of natural selection.

[4] Harvey PH, Bull JJ, Pemberton M, Paxton RJ (1982). The evolution of aposematic coloration in distasteful prey: A family model.. Am Nat.

[5] Harvey PH, Paxton RJ (1981). The evolution of aposematic coloration.. Oikos.

[6] Eisner T, Grant RP (1981). Toxicity, Odor Aversion, and Olfactory Aposematism.. Science.

[7] Cott HB (1940). Adaptive Coloration in Animals.

[8] Rothschild M (1961). Defensive odours and Müllerian mimicry among insects.. Trans R Entomol Soc Lond.

[9] Prudic KL, Noge K, Becerra JX (2008). Adults and Nymphs Do Not Smell the Same: The Different Defensive Compounds of the Giant Mesquite Bug (Thasus neocalifornicus: Coreidae).. J Chem Ecol.

[10] Eisner T, Eisner M, Seigler M (2005). Secret Weapons: Defenses of Insects, Spiders, Scorpions, and Other Many-Legged Creatures.

[11] Speed MP, Ruxton GD (2005). Warning displays in spiny animals: One (more) evolutionary route to aposematism.. Evolution.

[12] Gamberale-Stille G (2000). Decision time and prey gregariousness influence attack probability in naïve and experienced predators.. Anim Behav.

[13] Merilaita S, Ruxton GD (2007). Aposematic signals and the relationship between conspicuousness and distinctiveness.. J Theor Biol.

[14] Skelhorn J, Rowe C (2006). Avian predators taste–reject aposematic prey on the basis of their chemical defence.. Biol Let.

[15] Rowe C (1999). Receiver psychology and the evolution of multicomponent signals.. Anim Behav.

[16] Rowe C, Guilford T (1999). The evolution of multimodal warning displays.. Evol Ecol.

[17] Malakoff D (1999). Olfaction: Following the scent of avian olfaction.. Science.

[18] Steiger SS, Fidler AE, Valcu M, Kempenaers B (2008). Avian olfactory receptor gene repertoires: evidence for a well-developed sense of smell in birds?. Proc R Soc B.

